# Evaluation of UV-A and UV-B transmission through the windows of gas, hybrid, and electric vehicles

**DOI:** 10.1007/s00403-024-03771-x

**Published:** 2025-01-18

**Authors:** Grace E. Axelson, Jemima Constanza, Ryan Rezaee, Ava Axelson, Ally Cenci, Ava Khan, Rina Weimann, Erum N. Ilyas

**Affiliations:** 1 Department of Dermatology, Drexel University College of Medicine, 860 1St Avenue, Suite 8B, Philadelphia, PA 19406 USA; 2 UVtec, King of Prussia, PA USA; 3 Schweiger Dermatology, King of Prussia, PA USA

**Keywords:** Sun protection, Sun protective measures, Ultraviolet, Cars, Vehicles, Electric cars, Hybrid cars

## Abstract

UV-A exposure is a major risk factor for melanoma, nonmelanoma skin cancer, photoaging, and exacerbation of photodermatoses. Since people spend considerable time in cars daily, inadequate UV-A attenuation by car windows can significantly contribute to the onset or exacerbation of these skin diseases. Given recent market trends in the automobile industry and known impact of car windows on cumulative lifelong UV damage to the skin, there is a need to comparatively evaluate UV transmission across windows in electric vehicles (EV), hybrid vehicles (HV), and gas vehicles (GV) as well as variability based on year of manufacture and mileage to inform car manufacturers and consumers of the potential for UV exposure to the skin based on vehicle. To compare UV-A and UV-B transmission through EV, HV, and GV windows to evaluate differences in UV protection offered by various vehicle types. Comparative observational study that took place between June 10, 2024 and August 2, 2024. Outdoor setting with natural light exposure at car dealerships in Philadelphia, PA and New York, NY. 34 vehicles—15 gas vehicles (GV), 9 hybrid vehicles (HV), 10 electric vehicles (EV)—ranging from 2015 to 2025. Window status, with UV transmission measurements recorded with windows open and closed. UV-A and UV-B transmission through car windows was measured using UV transmission meters. The percent reduction in transmission was calculated. The front windshield and driver side window have statistically significant differences in UV-A attenuation across all vehicles with an average of 99.25% and 88.78% (p < 0.001), respectively. GV, HV, and EV all demonstrated significant differences in UV-A attenuation in most other vehicle windows compared to the front windshield. For GV, the front windshield, rear side windows (p = .176, p = .578) and back windshield (p = .457) blocked more UV-A than the front side windows. EV offered greater UV-A attenuation at the front and back windshield (p = .09) but not for any side windows, and HVs showed consistent differences in UV-A protection between the front windshield and all other windows. Domestic GV, trucks and luxury vehicles had no significant differences in UV-A attenuation across windows indicating reduced UV-A exposure for driver and passengers, whereas non-luxury vehicles had a notable difference in UV-A protection between the front windshield and all other windows. Regression analysis found mileage, not year of manufacture, to be a significant predictor of driver’s side UV-A attenuation, with more UV-A attenuation as vehicle mileage increases. Most vehicles evaluated offer effective UV-A and UV-B protection from the front windshield but lack sufficient UV-A protection for drivers nor consistently to other passengers with notable exceptions seen with domestic GV, trucks, and luxury vehicles. Mileage and not year of manufacture also contributed to additional UV-A attenuation. This underscores the importance of patient education on this known source for cumulative lifetime UV exposure and need for continued sun safety measures even while driving given potential UV-A impact on the skin.

## Introduction

Ultraviolet (UV) exposure is a major risk factor for melanoma and nonmelanoma skin cancer, in addition to photodamage and exacerbation of photosensitive skin disorders. Among other activities, individuals are frequently exposed to UV radiation when driving automobiles. A study by Singer et al. [17] found a correlation between subjects with more photodamage on their left side and the time they spent driving. In fact, research has shown that the left side of the head, neck, arm, and hand receive up to 6 times as much UV radiation as the right side in those sitting on the left side of the car (i.e., in the driver’s seat) [6]. Individuals in the United States (US) who drive motor vehicles have been noted to have increased incidence of skin cancer on the left side with an assumption that the differential exposure to UV is likely based on car window photo exposure [4, 6]. In further support of this theory, a study from Australia, where automobiles are driven on the opposite side of the road, found that drivers had an increased incidence of actinic keratosis and skin cancer on the right side of the face [9]. Photosensitive eruptions have been shown to be potentially triggered within 30 min of UV-A exposure through side windows made of tempered glass found in many cars [11]. UV-A rays are able to penetrate glass windows whereas UV-B rays cannot, therefore car windows may not offer adequate attenuation of UV-A rays.

A previous study involving cars manufactured up to 2014 by Boxer Wachler [4] found that only the front windshield, and not the driver’s side window, consistently protects the driver’s eyes and faces against UV-A rays with an average side window protection of 71%. The difference in protection afforded by car windows is thought to be related to the type of glass used, with windshields typically made of laminated glass, whereas side and rear windows are usually made of tempered glass [1]. While both block UV-B transmission, laminated glass blocks the majority of UV-A radiation while tempered glass does not [1].

While previous studies have evaluated UV-A and UV-B transmission through car windows, most were prior to the introduction and widespread use of electric vehicles. Electric vehicles may consider windows with more embedded technology or properties that protect against solar effects of heating the car from infrared radiation that can increase battery consumption [15]. These materials may indirectly contribute to added UV protection. Given recent market trends in the automobile industry and known impact of car windows on cumulative lifelong UV damage to the skin [13], there is a need to comparatively evaluate UV transmission across windows in electric vehicles (EV), hybrid vehicles (HV), and gas vehicles (GV) as well as variability based on year of manufacture and mileage to inform car manufacturers and consumers of the potential for UV exposure to the skin based on vehicle.

## Methods

In this comparative observational study, samples of EV, HV, and GV were evaluated. This study was determined to be exempted from institutional review board approval given the lack of human subjects involved. An online search was conducted to identify top sales of EV (Axios.com, Cars.com), HV (Axios.com, GreenCars.com), and GV (VisualCapitalist.com, Motor1.com) based on 2023 data with top five best-selling vehicles in each category selected. Two vehicles from the original list, the Rivian (EV) and Jeep Wrangler Hybrid (HV), were difficult to locate at nearby dealerships, so they were removed from the list and replaced by other car models that were more consistently available. Sixteen car models were evaluated, with two cars from each model to ensure accuracy, consisting of 10 EV, 9 HV, and 15 GV from model years 2015 to 2025. Only one Hyundai Ioniq 5 (EV) and one Toyota Sienna Hybrid (HV) were tested due to limited availability at local dealerships (Table 1).

**Table 1:** Cars used in study and corresponding sales rank based on 2023 data

	Gas Vehicles		Hybrid Vehicles		Electric Vehicles
1	Ford F-150	**1**	Honda CR-V Hybrid	**1**	Tesla Model Y
2	Chevrolet Silverado	**2**	Toyota Rav4 Hybrid	**2**	Tesla Model 3
3	RAM Pickup	**3**	Honda Accord Hybrid	**3**	Chevrolet Bolt
4	Toyota RAV4	**5**	Toyota Sienna Hybrid	**5**	Ford Mustang Mach-E
5	Honda CR-V	**12**	Ford Escape Hybrid*	**7**	Hyundai Ioniq 5*
15	Honda Accord**				

^*^Vehicle was not in the top 5 sales list and was chosen for inclusion in our study due to convenience over another vehicle that was originally included in the top 5 sales list

^**^Vehicle was included due to convenience at local dealerships

From June through July of 2024, data was gathered in an outdoor setting with natural sunlight exposure from local car dealerships in Philadelphia, Pennsylvania and New York City (boroughs included the Bronx, Manhattan, and Queens). A UV transmission meter was used to measure UV-A and UV-B transmission (SolarMeter 4.0 and SolarMeter 6.0, respectively) with the window open and window closed. The SolarMeter devices measure UV with a photodiode sensor to detect specific wavelengths of light by converting the energy of the UV light measured into an electrical signal to allow it to be quantified. The 4.0 device detects UV-A between wavelengths of 320 nm and 400 nm while the 6.0 device detects UV-B between 250 and 320 nm. Both SolarMeter devices were calibrated in reference to NIST standards by Solar Light Company, Inc within the past year. For the purposes of this study, we focused on percent reduction in UV transmittance through glass as this method of measurement proved convenient for testing a number of vehicles.

UV-A and UV-B transmission readings were recorded for each car window: front windshield, driver's side window, passenger window, rear windows, and back windshield. Measurements taken were evaluated in the context of percent reduction in UV transmission (i.e., percentage of UV blocked). Paired t-tests were done to evaluate for any statistically significant differences in mean UV attenuation between the front windshield and other car windows. Simple linear regression was also used to evaluate the relationship between the year the car was made and mileage of the car and UV-A attenuation of the driver’s side window. Statistical analysis was performed to compare the percent reduction in UV-A and UV-B transmission between various categories using IBM SPSS Statistics Version 29 0.2.0 (20).

## Results

The percent UV-A and UV-B attenuation for each vehicle window is displayed in Table 2. Percent UV-A attenuation across all vehicles ranged from 65.22 to 100%. The average UV-A attenuation from the front windshield was 99.25% with minimum UV-A attenuation of 91.67%. All windows demonstrated 100% UV-B attenuation except for the rear left window in the 2024 Honda CR-V (37.5%). Additional measurements were taken for this window to confirm the accuracy of our reading, but it continued to be an outlier.

**Table 2:** Data on Automobiles Tested. Percentages of UVA and UVB Attenuation Observed

	Automobile (Make/Model)	Year	Miles	UVA Attenuation (%)						UVB Attenuation (%)					
				Front	Driver's	Passenger	Rear Left	Rear Right	Back	Front	Driver's	Passenger	Rear Left	Rear Right	Back
Gas Vehicles (GV)	Ford F-150	2016	34 k	100	100	100	95.16	100	100	100	100	100	100	100	100
	Ford F-150	2018	193 k	100	100	100	100	100	100	100	100	100	100	100	100
	Ford F-150	2024	0	100	100	100	100	100	100	100	100	100	100	100	100
	Chevrolet Silverado	2021	82 k	98.41	80.49	86.96	100	100	100	100	100	100	100	100	100
	Chevrolet Silverado	2018	42 k	98.39	76.47	91.89	98.36	100	100	100	100	100	100	100	100
	Ram Pickup	2019	54 k	100	100	100	100	100	100	100	100	100	100	100	100
	Ram Pickup	2020	115 k	100	100	100	100	100	100	100	100	100	100	100	100
	Toyota Rav4	2016	116 k	98.39	92.5	89.29	95	95	93.55	100	100	100	100	100	100
	Toyota Rav4	2021	34 k	100	91.43	78	100	97.92	98.33	100	100	100	100	100	100
	Toyota Rav4	2022	29 k	97.5	73.53	91.3	100	100	100	100	100	100	100	100	100
	Honda CR-V	2022	25 k	97.22	78.43	88.24	97.92	100	91.67	100	100	100	100	100	100
	Honda CR-V	2024	1.7 k	100	72.41	84	100	100	93.75	100	100	100	37.5	100	100
	Honda CR-V	2019	117 k	91.67	100	97.3	76.92	85	97.22	100	100	100	100	100	100
	Honda Accord	2018	91 k	100	100	69.23	100	100	100	100	100	100	100	100	100
	Honda Accord	2023	19 k	97.78	73.08	81.48	92.59	96.15	95.56	100	100	100	100	100	100
Hybrid Vehicles (HEV)	Honda CRV Hybrid	2024	0	100	83.33	81.48	100	96.55	92.31	100	100	100	100	100	100
	Honda CRV Hybrid	2023	29 k	97.62	82.76	91.3	96	100	100	100	100	100	100	100	100
	Toyota Rav4 Hybrid	2019	5 k	100	80	90	96.23	100	92.31	100	100	100	100	100	100
	Toyota Rav4 Hybrid	2021	30 k	100	94.74	87.5	95.24	90	98.39	100	100	100	100	100	100
	Honda Accord Hybrid	2024	31.5 k	100	80	80	77.78	80	80	100	100	100	100	100	100
	Honda Accord Hybrid	2018	67 k	100	100	100	78.13	83.33	77.78	100	100	100	100	100	100
	Toyota Sienna Hybrid	2023	35.6 k	100	85.71	74.29	96.97	96.72	97.56	100	100	100	100	100	100
	Ford Escape Hybrid	2024	0	100	85.71	82.61	96.15	95.83	96.15	100	100	100	100	100	100
	Ford Escape Hybrid	2020	18 k	100	89.47	87.5	69.57	93.55	97.78	100	100	100	100	100	100
Electric Vehicles (EV)	Tesla Model Y	2022	49 k	100	100	100	100	100	100	100	100	100	100	100	100
	Tesla Model Y	2024	9 k	100	100	100	100	100	100	100	100	100	100	100	100
	Tesla Model 3	2019	31 k	98.39	81.25	78.95	94.74	79.69	98.39	100	100	100	100	100	100
	Tesla Model 3	2023	32 k	100	100	100	82.35	85.71	100	100	100	100	100	100	100
	Chevrolet Bolt	2023	16 k	100	84	81.25	83.87	79.41	85.19	100	100	100	100	100	100
	Chevrolet Bolt	2017	16 k	100	74.36	86.36	70.27	90	82.61	100	100	100	100	100	100
	Chevrolet Bolt	2020	19 k	100	65.22	87.5	77.27	94.74	97.37	100	100	100	100	100	100
	Ford Mustang Mach-E	2021	10.5 k	100	100	100	100	100	100	100	100	100	100	100	100
	Ford Mustang Mach-E	2024	3 k	100	83.33	86.21	100	96.3	100	100	100	100	100	100	100
	Hyundai Ioniq 5	2025	0	100	100	100	100	100	95	100	100	100	100	100	100

**Fig. 1:**
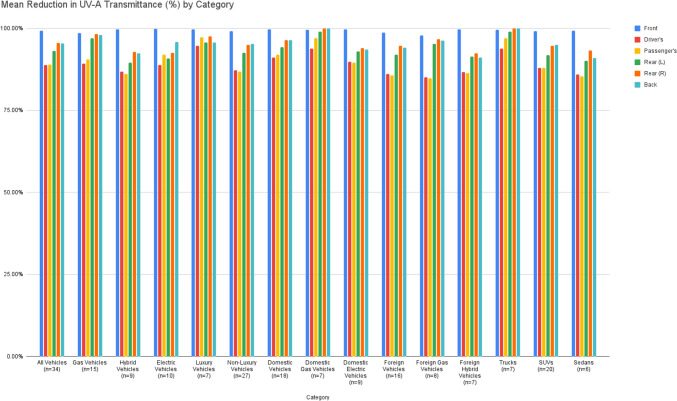
Mean reduction in UV-A transmittance (%) for each window by vehicle category

For all three vehicle types, the UV-A attenuation of the front windshield was statistically significantly different than nearly all other windows in the vehicle. (Table 3, Fig. 1) For GV alone, the front windshield generally provides different UV-A attenuation compared to the driver's side and passenger side windows, but not compared to the rear windows (p = 0.176, p = 0.578) and back windshield (p = 0.457). For EV, analysis suggests that the front windshield generally provides different UV-A attenuation compared to all other windows except for the back windshield (p = 0.09). Lastly, findings for HV suggest that the front windshield generally provides different UV-A attenuation compared to all other windows (p < 0.05).

**Table 3:** Mean Reduction in UVA Transmittance by Category

	Mean reduction in UVA transmittance (%)					
	Front	Driver's	Passenger's	Rear (L)	Rear (R)	Back
All vehicles *(n* = *34)*	99.25%	88.78%*	88.99%*	93.18%*	95.57%*	95.36%*
		< *0.001*	< *0.001*	< *0.001*	*0.002*	*0.003*
Gas vehicles (GV) *(n* = *15)*	98.62%	89.22%*	90.51%*	97.06%	98.27%	98.01%
		*0.008*	*0.007*	*0.176*	*0.578*	*0.457*
Hybrid vehicles (HEV) *(n* = *9)*	99.74%	86.86%*	86.08%*	89.56%*	92.89%*	92.47%*
		< *0.001*	< *0.001*	*0.028*	*0.025*	*0.033*
Electric vehicles (EV) *(n* = *10)*	99.84%	88.82%*	92.03%*	90.85%*	92.58*	95.85%
		*0.024*	*0.018*	*0.035*	*0.020*	*0.090*
Luxury *(n* = *7)* *Ram Pickup, Tesla Model Y, Tesla Model 3, Honda Accord Hybrid (Touring)*	99.66%	94.68%	97.33%	95.76%	97.62%	95.73%
		*0.178*	*0.185*	*0.248*	*0.439*	*0.275*
Non-luxury *(n* = *27)*	99.15%	87.25%*	86.82%*	92.51%*	95.04%*	95.27%*
		< *0.001*	< *0.001*	*0.002*	*0.003*	*0.007*
American cars *(n* = *18)*	99.69%	91.17%*	91.93%*	94.21%*	96.4%*	96.45%
		< *0.001*	*0.001*	*0.023*	*0.042*	*0.064*
*Gas* *(n* = *7)*	99.54%	93.85%	96.98%	99.07%	100%	100%
		*0.175*	*0.191*	*0.562*	*0.172*	*0.172*
*Electric* *(n* = *9)*	99.74%	89.88%*	89.52%*	92.94%*	93.99%	93.58%
		*0.009*	*0.012*	*0.049*	*0.064*	*0.073*
Foreign Cars (n = 16)	98.76%	86.09%*	85.68%*	92.02%*	94.64%*	94.14%*
		< *0.001*	< *0.001*	*0.016*	*0.025*	*0.025*
*Gas* *(n* = *8)*	97.82%	85.17%*	84.85%*	95.30%	96.76%	96.26%
		*0.029*	*0.012*	*0.237*	*0.368*	*0.320*
*Hybrid* *(n* = *7)*	99.66%	86.65%*	86.37%*	91.48%	92.37%	91.19%
		*0.004*	*0.007*	*0.065*	*0.064*	*0.054*
Trucks *(n* = *7)*	99.54%	93.85% *0.175*	96.98% *0.191*	99.07% *0.562*	100% *0.172*	100% *0.172*
SUVs *(n* = *20)*	99.12%	88%* < *0.001*	88.02%* < *0.001*	91.84%* *0.006*	94.66%* *0.004*	94.96%* *0.012*
Minivan *(n* = *1)*	100%	85.71%	74.29%	96.97%	96.72%	97.56%
Sedans *(n* = *6)*	99.23%	85.97%* *0.029*	85.34%* *0.026*	90.12% *0.080*	93.25% *0.191*	90.94% *0.111*

^*^Indicates *p* < 0.05

Both foreign and domestic vehicles statistically showed there is a significant difference in UV-A attenuation between the front windshield and the other windows of the car (p < 0.05), except there was no difference found between the front and back windshields in domestic cars (p = 0.064). When separated by fuel type, domestic GV demonstrated no significant difference in UV-A attenuation between any of the windows (p > 0.05 for all). Both foreign GV and HV showed a statistically significant difference in UV-A attenuation between the front windshield and the driver’s and passenger’s side windows (p < 0.05), but no difference was found between the front windshield and other windows of the car (p > 0.05).

Trucks showed no notable variation in UV-A attenuation between the front windshield and all other windows of the car (p > 0.05 for all), indicating similar UV-A attenuation across all windows. In contrast, the SUVs demonstrated a significant difference in UV-A attenuation between the front and all other car windows (p < 0.05 for all) and sedans only for the drivers and front passenger side windows. Minivans could not be evaluated for variations due to small sample size.

Luxury vehicles also showed no significant difference in UV-A attenuation between the front windshield and all other windows of the car (p > 0.05 for all), suggesting UV-A attenuation comparable to that of the front windshield for all windows. In contrast, non-luxury vehicles demonstrated a significant difference in UV-A attenuation between the front and all other car windows (p < 0.05 for all).

Linear regression analysis found mileage to be a significant predictor of driver’s side UV-A attenuation (adjusted R^2^ = 0.151, standardized β = 0.42, p = 0.013). This suggests 15.1% of the variance in driver’s side UV-A attenuation can be explained by mileage. For every one standard deviation increase in mileage, UV-A attenuation increases by 0.42 standard deviations. Year of manufacture was not found to be a significant predictor of driver’s side UV-A attenuation (p = 0.077). Caution should be taken when considering the results of the regression analysis as our data was not perfectly linear.

## Discussion

According to the AAA Foundation for Traffic Safety’s American Driving Survey in 2022, drivers reported spending an average of 60.2 min per day driving with those between the ages of 35 to 49 at 78.3 min daily [2]. This is a significant increase from prior years. With men reporting substantially more time driving each day compared to women, the increased incidence of left-sided skin cancer with UV exposure while driving was also most notable in men suggesting a cumulative impact of UV exposure over time [2, 17]. Overall, a significant difference was found in UV-A protection offered by the front windshield compared to the driver’s and passenger’s side windows for all cars evaluated, suggesting that drivers and passengers are still not well protected from UV-A while driving from the car windows. It should be noted that despite the difference there has been an increase in mean UV-A attenuation across all cars to 88.78% (range 72–100% [95% CI, 79.2–98.3%) compared to 71% average (range, 44–96% [95% CI, 66.4–75.6%]) in 2014 [4]. Further, the difference in mean UV-A attenuation between the front windshield and driver’s side window was 10.47% (95% CI, 7.1–13.84% [p < 0.001]) compared to 25% (95% CI, 21–30% [p < 0.001]) in 2014 [4].

For GV in particular, there were statistically significant differences noted in comparing UV-A attenuation of driver and passenger side windows to the rear side windows suggesting that rear side windows offer comparable UV-A attenuation to the front windshield. It is unclear why EV and HV would not offer similar benefits for the rear side windows. We theorize that the variation in UV-A transmission between the front side windows and the rear side windows may be the result of color and thickness variations of the glass utilized however this could not be confirmed on visual inspection. UVR transmission can vary depending on the type of window glass utilized within the vehicle and is also influenced by the color and thickness of the glass [14, 18].

**Fig. 2:**
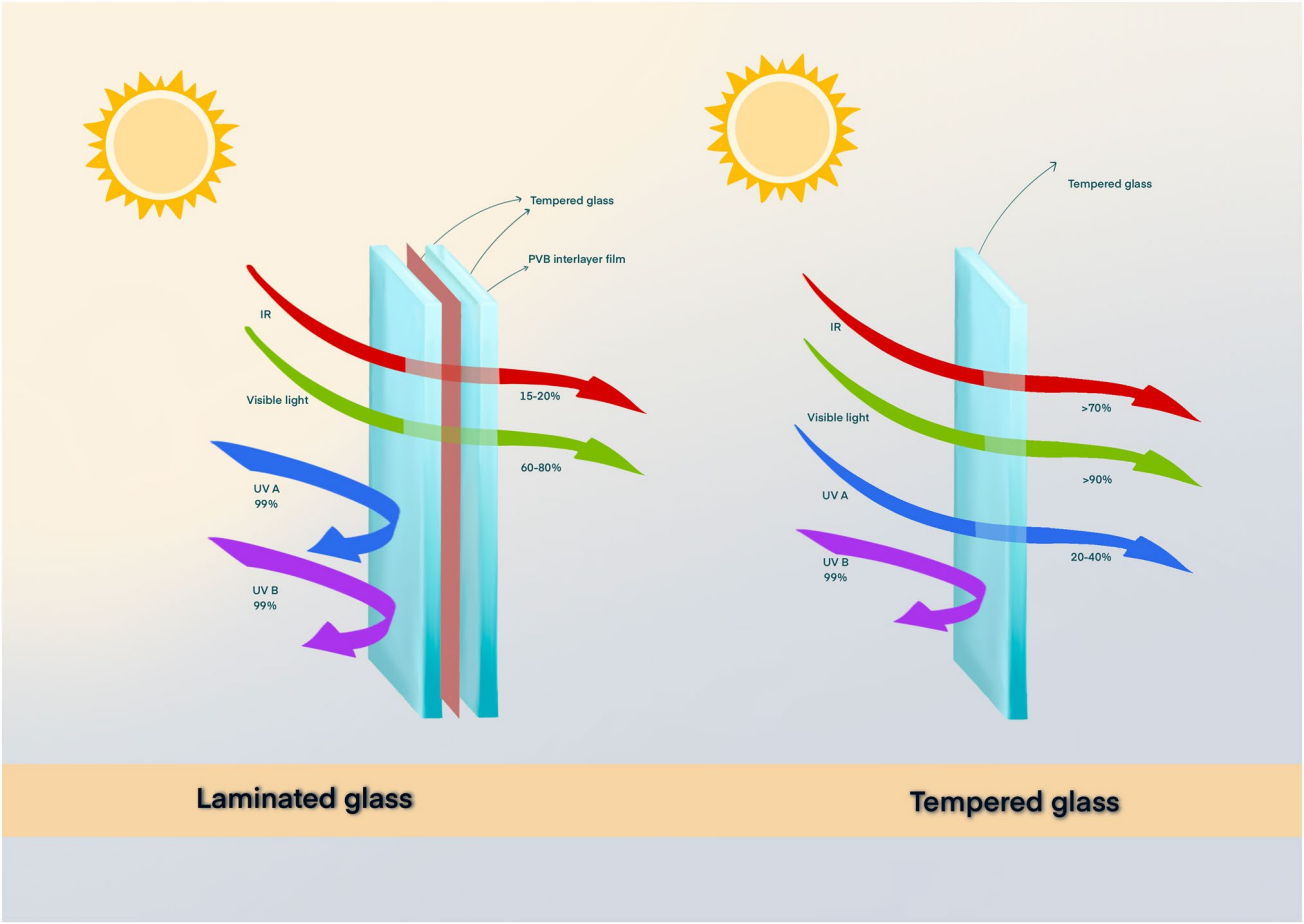
Demonstration of penetration of infrared (IR), visible light, UV-A, and UV-B emitted from sun through laminated and tempered glass

Commonly, vehicle windows utilize either tempered or laminated glass types, with stark differences in UVR transmission between the two forms [1, 15] (Fig. 2). Laminated glass is more effective at blocking UV-A rays [1, 14, 18]. Both glass styles are formed from annealed glass, which is created by the melting and slow cooking of silica, salt, limestone, dolomite, feldspar, and soda ash [1]. Tempered glass is created by the rapid cooling following formation of the glass and is defined by its small fragmentation pattern that follows shattering to avoid injury; dually, tempered glass is four times stronger than annealed glass [1]. Typically used in side and rear car windows, tempered glass has similar UVR transmission to basic annealed glass, blocking almost all UV-B radiation, but blocking only roughly 21% of UV-A radiation which may be responsible for the asymmetric UV related changes to the skin noted [3]. In contrast, laminated glass is formed by adding a layer of polyvinyl butyral (PVB) between two or more layers of glass, giving the window high levels of strength that typically prevent shattering that make it an ideal choice for front windshields in automobiles [1]. Additionally, laminated glass blocks almost all UVR, allowing only 2% transmission of UV-A radiation. Cost may be a limiting factor preventing the use of laminated glass in all car windows. Additionally, while laminated glass can prevent break-ins due to the effort required to break them and may offer better safety in collisions, some manufacturers and consumers may be strayed away from the use of laminated glass in all car windows due to safety and difficulty breaking windows in emergency situations requiring one to escape from the vehicle.

Interestingly, although EVs and HVs could theoretically benefit from windows or window treatments that block or reduce infrared solar radiation—thereby preserving battery consumption from air conditioning and potentially incidentally lowering UV exposure [7]—data from this study did not support this finding. For EV, there is a statistically significant difference in UV-A attenuation between the front windshield and the driver's side, passenger side, rear left, and rear right windows and no statistically significant difference in UV-A attenuation between the front windshield and the back window in cars evaluated. For HV evaluated, there is a statistically significant difference in UV-A attenuation between the front windshield and all of the other windows including the back windshield (driver's side, passenger side, rear left, rear right, and back windshield).

Domestic GV demonstrated comparable UV-A attenuation in all car windows. This was not observed in foreign GV. This suggests that domestic GV offers superior UV-A protection for drivers and passengers compared to foreign GV. This variation could not be explained by differences in safety regulations applied to car windows in the U.S. compared to other countries, as these regulations should apply to manufacturers selling vehicles in the U.S. US regulations state that the windshields and all side windows must allow a minimum of 70% of visible light transmission to ensure safe driving standards and ability to utilize auxiliary mirrors properly [1]. Domestic vehicles across all types (GV, HV, EV) did not offer comparable UV-A attenuation across all windows making it unclear why this advantage was limited to the domestic GV category.

Vehicles designated as “trucks” only require 70% transmission of visible light through the windshield and front driver and passenger windows, and do not have a regulatory threshold for the rear row windows which allows for different colors of window glass (green, dark and light gray) to be utilized that prevent higher levels of UV-A transmission into the vehicle [1, 19]. Given that the front windshields can consistently protect against UV-A transmission, added tinting and decreasing visibility through windows is not the only means to contribute to UV-A attenuation. Data from the vehicles evaluated in this study demonstrated that trucks (Ford F-150, Chevrolet Silverado, Ram Pickup) offered comparable UV-A attenuation in all windows suggesting better UV-A protection for driver and passengers compared to the SUVs, minivans, and sedans evaluated.

**Fig. 3:**
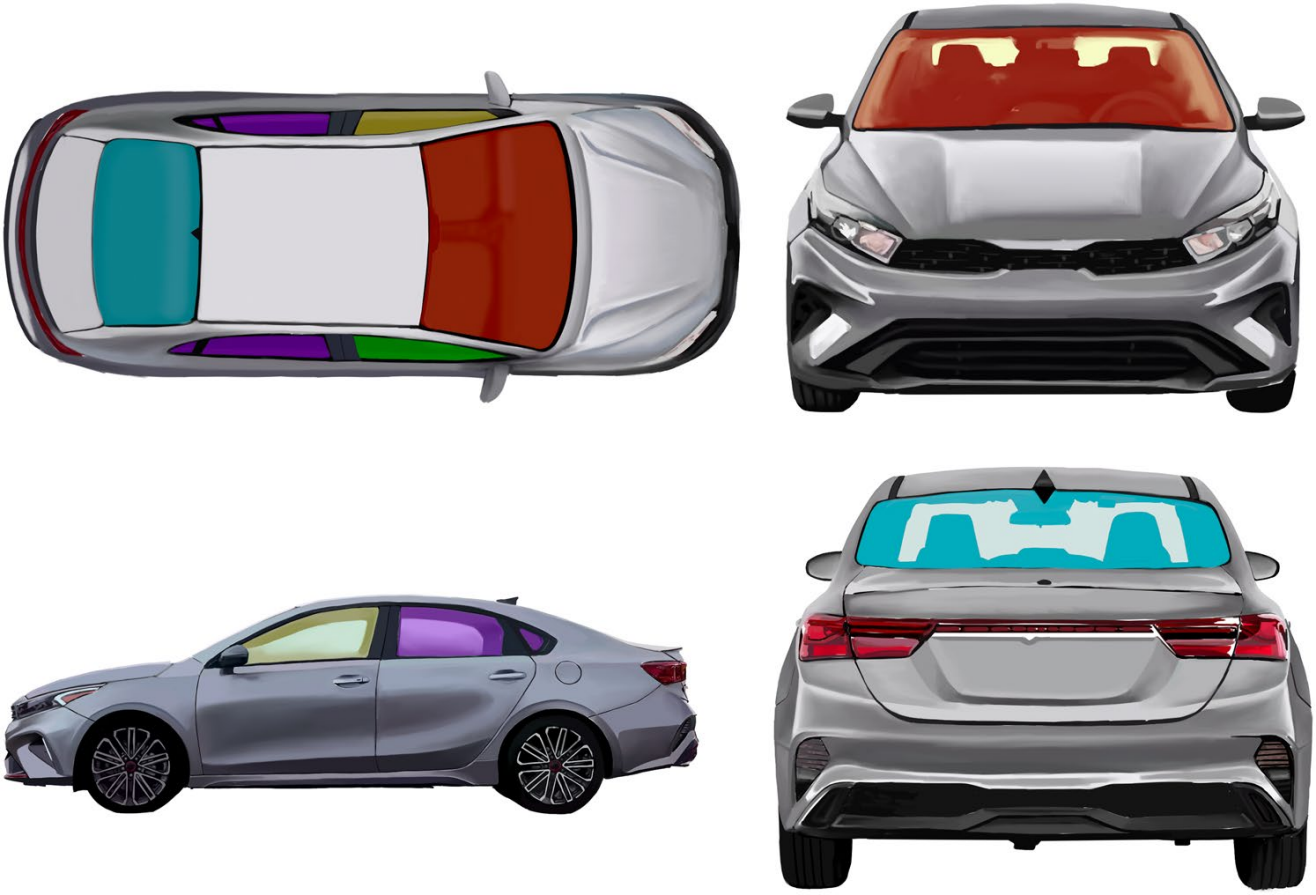
Diagram of windows evaluated including front windshield (red), rear windshield (blue), driver’s side window (yellow), front passenger window (green), and rear passenger windows (purple)

Aside from the domestic GV and trucks category, luxury vehicles were found to have comparable UV-A attenuation in all car windows, offering better UV-A protection for the driver and all passengers (Fig. 3). A prior study evaluating the cost of cars and the UV-A exposure through windows did not find a correlation between UV-A attenuation and cost, however this study included both luxury cars and expensive sports cars [5]. It was theorized that luxury cars may use acoustic glass for side windows for noise reduction and soundproofing which is laminated and may also have added thickness [16].

Regression analysis found mileage, not year of manufacture, to be a significant predictor of driver’s side UV-A attenuation, with more UV-A attenuation as vehicle mileage increases. This may be due to increased exposure to road and environmental contaminants accumulating on vehicles with use over time. The interaction of car surfaces with rain and road spray has been noted to degrade the vision of drivers [10]. Washer fluid applied to the front screen can be carried on to the side glass, where it may contribute to compromised visibility through the side glass and to the door mirror [10]. Exterior water management, particularly for the front side glass, remains a main focus for manufacturers [10].

As rideshare apps (e.g., Uber and Lyft) are increasingly being used, it becomes important to consider UV exposure while in the rear passenger seats. While taking into account both employee and customer safety, these findings may push rideshare companies towards adopting sun-safe practices. This may include implementing a sun safety training to increase awareness of this occupational UV hazard such as introducing recommendations for drivers to keep car windows closed and encouraging consideration to choosing cars with windows that have been shown to be UV protective or consideration to protective window films.

Window films blocking UV-A and infrared radiation may be added to car windows [1, 18]. Studies have shown that the addition of window films to tempered glass, which is commonly used in side and back windows, can result in more than 99% reduction of UVR transmission [3, 8]. This is particularly useful for patients with photodermatoses, although may not be necessary in all vehicles based on the data associated with domestic gas cars and luxury cars. Consideration to local laws and regulations regarding minimal visibility through windows must be factored in before discussing recommendations regarding UV risk reduction strategies with patients.

There are limitations to this study that should be noted. Variations in car models and window tinting may affect the study results. Unfortunately, information regarding replacement windows and post purchase treatments to windows was not readily available nor obvious upon car inspection, nor was information on the type of window glass used in each car window. Another limitation to this study included variations in environmental factors such as weather conditions that may influence UV transmission measurements. To minimize this risk, percent reduction in UV transmission through car window glass was calculated and used as our primary outcome measure for standardized comparison. Additionally, a study by Huang and Chalmers [12] demonstrated that the SolarMeter devices yield acceptable values that are within ± 10% when compared to those obtained from a spectroradiometer; however, it is important to note that our primary focus is on percent reduction rather than absolute values. Another limitation is the small sample size. Only 34 cars were included in our study, with ≤ 15 in each fuel type category and other subcategories used in our analysis. The variations noted between cars of the same make and model were challenging to explain aside for possible differences in sourcing materials during manufacturing or post-production modifications made by consumers. Lastly, our study does not evaluate the differences in UV exposure between casual and professional drivers, which warrants further research to better understand potential variations in health outcomes due to differing amounts of time spent in vehicles. Future studies should include a larger sample size and deeper analysis comparing brands individually to support conclusions drawn from statistical analysis.

## Conclusion

This study provides valuable insight into the level of UV protection offered by vehicle windows based on vehicle type over the past decade. Our data highlights current evidence that most vehicles evaluated offer effective UV-A and UV-B protection from the front windshield but do not offer sufficient UV-A protection to the driver’s side nor consistently to other passengers with notable exceptions seen with domestic gas vehicles, trucks, and luxury vehicles. Mileage and not year of manufacture also contributed to additional UV-A attenuation. This data underscores the importance of patient education on this known source for cumulative lifetime UV exposure and need for continued sun safety measures even while driving given potential UV-A impact on the skin. It also informs manufacturers on consideration of window type for consumer UV safety.

## Data Availability

All data is provided within the manuscript.
